# Curcumin as adjuvant therapy to improve remission in myeloma patients: A pilot randomized clinical trial

**DOI:** 10.22088/cjim.13.2.9

**Published:** 2022

**Authors:** Damai Santosa, Catharina Suharti, Ignatius Riwanto, Edi Dharmana, Eko Adhi Pangarsa, Budi Setiawan, Suyono Suyono, Mika Lumban Tobing, Suhartono Suhartono, Soeharyo Hadisapurto

**Affiliations:** 1 Division of Hematology-Medical Oncology, Department of Internal Medicine, Faculty of Medicine, Diponegoro University/Dr. Kariadi General Hospital, Jawa Tengah, Indonesia; 2 Departmen of Surgery, Faculty of Medicine, Diponegoro University, Jawa Tengah, Indonesia; 3 Department of Parasitology, Faculty of Medicine, Diponegoro University, Jawa Tengah, Indonesia; 4 Department of Environmental Health, Faculty of Public Health, Diponegoro University, Jawa Tengah, Indonesia; 5 Division of Topical and Infectious Disease, Department of Internal Medicine, Faculty of Medicine, Diponegoro University/Dr. Kariadi General Hospital, Jawa Tengah, Indonesia

**Keywords:** Myeloma, Overall remission, NF-κB, VGEF, IL-6, TNF-α

## Abstract

**Background:**

The treatment for ineligible transplant multiple myeloma is melphalan prednisone. Curcumin has an anti-inflammatory and antiangiogenesis in cancer-directed to nuclear factor-kappa B (NF-kB) pathway. Interleukin 6 (IL-6), vascular endothelial growth factor (VEGF), tumor necrosis factor-alpha (TNF-α), C-reactive protein (CRP), and lactate dehydrogenase (LDH) were also involved in the pathogenesis of myeloma. No clinical study has evaluated the efficacy of curcumin in myeloma patients. To evaluate the efficacy of curcumin as adjuvant into melphalan prednisone in myeloma patients

**Methods:**

33 myeloma patients at Dr. Kariadi General Hospital, Semarang, Indonesia during 2016-2017 were randomly assigned single-blindedly into MPC (n=17) and control group (n=16). The MPC group was treated with melphalan 4 mg/m2, prednisone 40 mg/m2 for 7 days, and curcumin 8 gram daily for 28 days. The MP control group was treated with melphalan, prednisone, and placebo. The primary endpoint was the overall remission. Pre- and post-treatment was examined for NF-κB, VEGF, TNF-α, IL-6, LDH, and CRP levels All data analyses were per protocol.

**Results:**

There was a significant difference in overall remission between the MPC and MP control groups [75%vs 33.3%, x^2^=6.89, P=0.009]. A significant decrease of NF-κB, VEGF, TNF-α levels were shown in the MPC group compared with the MP control group. There was a significant decrease in IL-6 levels in a subgroup analysis of the MPC group. TNF-α levels had a significant correlation with remission [OR=1.35; (95%CI=1.03-1.76); P=0.03].

**Conclusion:**

Curcumin has an efficacy in improving overall remission and decreasing NF-κB, VEGF, TNF-α, and IL-6 levels in myeloma patients**.**

Multiple myeloma (MM) is the 2^nd^ most common hematologic malignancy after lymphoma with an estimated 24,2802 to 30,330 new cases and 12,650 deaths with the estimated world-wide 5-year prevalence which is approximately 230,000 patients. (1),(2) The therapy varies from chemotherapy, autologous bone marrow transplant (ABMT) until novel agents. (3) Combined complete or partial response rates and the near-complete or complete response rates were 47.6% and 7.2 %, respectively for therapy with melphalan prednisone (MP). The 2-year event-free survival rates were 27% for MP (4). Curcumin is a natural product derived from *Curcuma longa*, a member of the ginger family, Zingiberaceae. Curcumin has a molecular weight of 368.4, with the molecular structure of C21H20O6. In Indonesia, curcumin is very famous used as one of the spices and also as traditional medicine (5, 6). Curcumin has effect as an anti-inflammatory and anti-tumors as demonstrated in vitro and in vivo studies. Moreover, curcumin affects the progression of myeloma cell (7-11).

This interesting result make many researchers develop the studies of curcumin as a chemotherapeutic agent. Curcumin has less toxicity and its safety has been approved by the FDA (12). The clinical study of efficacy curcumin has proven to inhibit the progression of monoclonal gammopathy of undetermined significance (MGUS) as well as smoldering multiple myeloma (SMM) (13-15). Curcumin has an anti-inflammatory and anti-angiogenesis in cancer-directed to the target of the NF-κB pathway (16, 17, 18). The other parameters in disease activity and cytokine involved in the pathogenesis of myeloma were IL-6, VEGF, TNF- α, CRP, and LDH (19, 20). The primary outcome of this study was to prove the efficacy of curcumin in the improvement of the remission in myeloma patients. The secondary outcome was to evaluate the effect of curcumin on transcription factor and cytokine levels, including NF-κB, IL-6, VEGF, TNF-α, CRP, and LDH. 

## Methods

The study was organized at Dr. Kariadi Hospital, Semarang, Indonesia during 2016-2017. Inclusion criteria were as follows: new MM patients, aged over 18 years old, and ineligible transplant. The exclusion criteria were sepsis, severe infection, pregnancy, patients with severe disease (such as acute hepatitis, chronic hepatitis, cirrhosis) or elevation of aspartate aminotransferase (AST) >3 times upper limit normal (ULN), subjects who participated in another study, and poor performance status. The sample size calculation was 32 participants, a total of 16 patients in each group. The α and β errors chosen for these calculations were 0.05 and 0.2, respectively. The power was 80%. Patients with clinically suspected myeloma were evaluated for full blood count, albumin, globulin, urea, creatinine, calcium, protein electrophoresis, immunotyping, bone survey, bone marrow aspiration, and -2 microglobulin. The staging was classified according to the International Staging System (ISS). The diagnosis of myeloma was established according to the IMWG 2014 (21, 22). 

**Figure 1:**
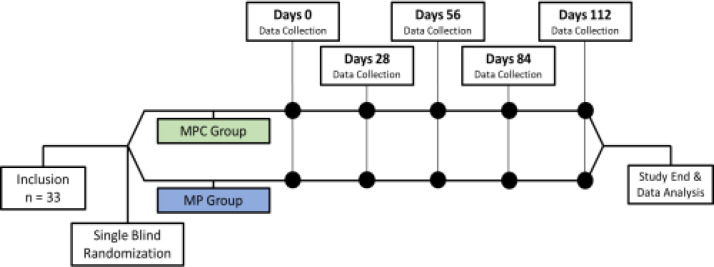
Randomized, single-blind & parallel design of the study. Multiple myeloma patients were single-blindedly randomized to MPC (green) and MP+Placebo (blue) groups. Data collection including full blood count (FBC), urea, creatinine, NF-κB, IL-6, CRP, LDH, VEGF, were collected in the baseline and every cycle (28 days)

Note: R: randomization, MPC: Melphalan dose; 4 mg/m2, Prednisone; 40 mg/m2 (day 1-7); Curcumin dose 8 mg/day (Day 1-28), treatment duration of 1 cycle for 28 days; total treatment 4 cycles; MP: Melphalan + prednisone + placebo

For the randomization, the participants were randomized into the group with a sealed envelope. A total of 33 patients were allocated randomly in two parallel study groups (Figure 1). The MPC group (17 patients) was treated with MP regimen (melphalan 4mg/m2, prednisone 40mg/2, for 7 days) and curcumin 8 gram /daily for 28 days. The control group (16 patients) was treated with MP regimen and placebo. Oral melphalan, prednisone was taken as a single dose. Curcumin was taken orally – 4 caplets, twice daily. Curcumin 1000 mg (BCM 95, Biocurcem ®) is composed of curcuminoid complex 95% and essential oils from turmeric (23) purchased from PT. SOHO Global Health, Indonesia. Each patient was followed up every 28 days, for a total of 4 cycles. The checklist was used for data collection and was filled in each visit separately for every subject. The contents of the checklist were the patients' profiles (age, sex, education level), and laboratory data, including full blood count (FBC), urea, creatinine, NF-B, IL-6, CRP, LDH, VEGF, and patient group (treatment or control). The physical examination of the patients was performed by a physician in every visit (single blindness). Remission status, NF-B, IL-6, CRP, LDH, VEGF, and TNF- were evaluated after the end of the study.

The blood was taken during office hours from the vein and collected in a tube that contains 10 ml EDTA. The blood was centrifuged, then the plasma was collected and kept under -80^◦^C Refrigerator at GAKI Laboratory, UNDIP Semarang. NF-κB, IL-6, VEGF, and TNF-α levels were measured by ELISA following the manufacturer´s instruction in GAKI Laboratory, UNDIP. CRP and LDH levels were measured at Dr. Kariadi Hospital. Protein M serum and FLC were measured in the Cito laboratory and Prodia laboratory, respectively. Complete blood count (CBC) was measured by flow cytometry method (Cell-Dyn Saphire; Abbott Diagnostics Division, Santa Clara, CA, USA). Serum calcium, CRP, and plasma albumin was measured by homogenous enzymatic colorimetric assay (Dade Behring; Siemens, German). Immunofixation electrophoresis is used to identify the clonality (type) of M-proteins measured by agarose gel. NF-κB (p56 /total) levels were measured by Enzyme-Linked Immunosorbent Assay (ELISA) Kit test, Human. VEGF serum levels were measured by the VEGF ELISA test kit (Quantikine®, Human Soluble VEGF R2 Immunoassay, Catalog Number DVRE 200). IL-6 serum levels were measured by the ELISA kit test (Human IL-6 Immunoassay Catalog Number HS600B). *TNF-α* serum levels were *measured* by human *TNF-α*  (ELISA) test kits. The remission was evaluated by bone marrow aspiration, the level of M protein or FLC (free light chain) was classified according to EBMT criteria (for common myeloma type) and IMWG criteria (for light chain myeloma) (24-26). 

BCM-95^®^CG (Biocurcem™) 1000 mg capsules were supplied by Soho Global Health. Biocurcem ^TM ^contains Curcuma longa rhizome (Curcuminoid complex 95%) with high bioavailability.

**Ethical approval and informed consent: **This clinical trial has been registered on the ISRCTN registry with study ID ISRCTN14131419. The protocol of the study was approved by the Ethics Committee of the Faculty of Medicine, Diponegoro University, and Dr. Kariadi General Hospital. This study was conducted following the Declaration of Helsinki and all the participants have signed the informed consent.

**Statistical Anal** **y** **sis** **: **The analysis within groups was performed using the Friedmann test. The analysis of nominal data between groups was done using the chi-square. The analysis of ratio data between groups was done using Mann-Whitney tests or independent t-test. The ratio data was conversed as delta () by comparison with baseline data. Correlation of variable was done using multiple logistic. The program of SPSS IBM Version 21 was used to analyze the data. The result of the analyzed data was considered significant if p<0.05.

## Results

**Demography and Characteristics Study: **During February 2016 - May 2017, there were 35 myeloma patients in Dr. Kariadi General Hospital, Semarang. Two subjects were excluded from the study. A total number of 33 patients were allocated randomly into two groups. The MPC and MP control groups consist of 17 subjects and 16 subjects, respectively. Four patients in the treatment group died due to sepsis, anemia, and thrombocytopenia in the first-month treatment. Three subjects in the control group died because of sepsis, melena, and anemia in the first-month treatment. One patient from the control group discontinued the study because of flushing after taking medication. One patient in the treatment group was lost of contact in the second month. One patient in the control group died because of anemia and sepsis in the third-month treatment. 

**Figure 2:**
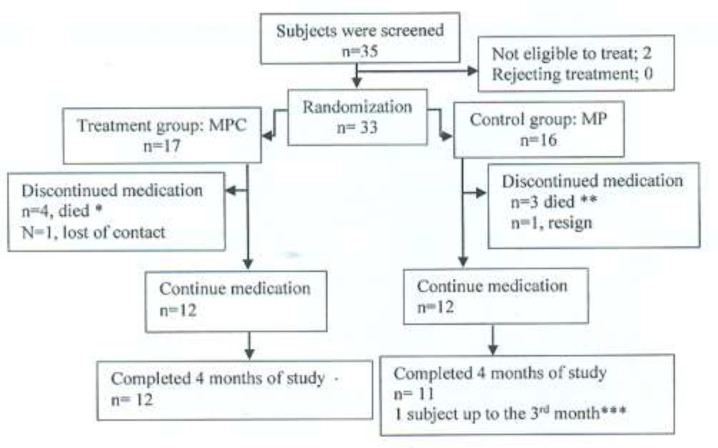
Consort of study Description: MPC: melphalan, prednisone, curcumin MP; melphalan, prednisone, placebo. Four patients in the treatment group died because of sepsis, anemia, and thrombocytopenia in the first-month treatment. ** Three patients in the control group died because of sepsis, melena, and anemia in the first-month treatment. One patient in the control group stopped taking medication because of flushing. One patient in the treatment group was lost of contact in the second monthly treatment. *** One patient in the control group died because of anemia and sepsis in the third-month treatment

Table 1 showed the baseline characteristics of the study population. There were no significant differences in the demographics and characteristics of the study population (age, BMI, creatinine, β2m, albumin, calcium, gender, bone lytic lesions, fractures, myeloma type, anemia, the percentage of the plasma cell, stage, and performance status). The consort of the study was shown in Figure 2.

**Effect curcumin in the remission: **The overall remission in the MPC group was better than the control. Table 2 showed the overall remission (OR) of myeloma patients treated with melphalan, prednisone, and curcumin increased to 75.0 %, compared with the MP control group of 20.0%. This result was statistically significantly different with *p*<0.05 (X^2^= 6.89, df=1, *P*=0.01). The effect of curcumin in the transcription factor and cytokine levels was shown in table 3. The decrease of NF-κB levels in the MPC group was higher than the MP control group. The table demonstrated the trend of reduction of NF-κB levels that increased in the MPC group. In the 4^th^ month, the reduction of NF-κB levels reached a significant decrease significantly in the MPC group compared with control (0.66 vs-0.05, *P*=0.027). The trend reduction of IL-6 increased in the MPC group, and was analyzed with the Friedman test. The reduction of IL-6 level showed a significant decrease in the MPC group (*P*=0.00), not in the MP group (*P*=0.19). However, there was no significant difference between the two groups in IL-6 levels. There was significant decrease of VEGF levels in the MPC group compared with MP control [(month 1, 100.20 vs 18.87; *P*=0.021), (month 2, 76.46 vs-80.82; *P*=0.013), (month 3, 76.04 vs 3.89; *P*=0.021), (month 4, 187.4 vs -43.38; *P*=0.001)]. The decrease of VEGF levels in the MPC group was better than the MP control group. The decrease of TNF- α was higher in the MPC group compared with the MP control group. This reduction of TNF-α levels was statistically significantly different (13.7 vs 2.07; *P*=0.004). However, there was no significant difference in CRP levels between the MPC and MP control groups. Analysis within-group showed a decrease of CRP levels in both MPC (*P*=0.01) and MP control group (*P*=0.04). There was a significant difference in LDH levels between the MPC and control groups. The trend of LDH level increased in the MPC group, but decreased in the control group (28.77 vs 75.41; *P*=0.008). 

**Multiple logistic regression: **The variable that correlated with remission with [*p≤ 0.25*] was included in the multivariate analysis model. Multiple logistic regression analysis showed in Table 4. These data demonstrated the correlation between TNF-α levels and remission [OR=1.35; (95%CI=1.03-1.76); *P*=0.03].

**Table 1:** Demography and Characteristics of the study population

**Characteristics**	**MPC** **Group**	**MP Control** **Group**	***P***
Subject (n=33)	17	16	
Age Mean Range Mean Rank	54.88± 10.23 36-77 13.9	59.13± 9.76 31-74 20.3	0.58 ^1^
Creatinine (mg/dL, *mean±SD*)	2.28±1.9	1.81±1.2	0.46 ^2^
β2 macroglobulin (mcg/L, *mean±SD*)	10.78±6.7	13.46±11.0	0.48 ^2^
Albumin (g/l, *mean ±SD*)	2.7±0.68	2.8±0.79	0.68 ^2^
Calcium (mmol/l, *mean ±SD*)	2.4±0.59	2.4±0.43	0.73 ^2^
Sex, [*n *(%)] Man Woman	12 (70.6) 5 (29.4)	8 (50.0) 8 (50.0)	0.23^ 3^
Lytic Lesion of the Bone [*n *(%)] Yes No	15 (88.2) 2 (11.8)	16 (100) 0	0.18 ^3^
Fracture Yes No	5 (29.4) 12 (70.6)	4 (25) 12 (75)	0.78 ^3^
Myeloma Sub-Type [*n *(%)] Immunoglobulin-G Immunoglobulin-A Immunoglobulin-M * Light chain *	7 (41.2) 4 (23.5) 2 (11.8) 4 (23.5)	7 (43.8) 5 (31.3) 1 (6.3) 3 (18.8)	0.91 ^3^
Anaemia [*n *(%)]	12 (100)	12 (100)	
Creatinine Clearance ≥ 60 ml/min < 60 ml/min	7 (41.2) 10 (58.8)	2 (12.5) 14 (87.5)	0.07 ^3^
Durie-Salmon Staging [*n *(%)] I II IIIA IIIB	0 3 (17.6) 6 (35.3) 8 (47.1)	0 0 9 (56.3) 7 (43.8)	0.17 ^3^
International Staging System [*n *(%)] I II III	1 (5.9) 1 (5.9) 15 (88.2)	1 (6.3) 1 (6.3) 13 (81.3)	0.99 ^3^
Hypercalcemia [*cut point=2,55mmo/L;* *n *(%)] Yes No	7 (41.2) 10 (58.8)	3 (18.8) 12 (75)	0.20 ^3^
Performance Status (ECOG) * [*n *(%)] 0 1 2 3	8 (66.7) 3 (25.0) 0 1 (8.3)	4 (33.3) 4 (33.3) 2 (16.7) 2 (16.7)	0.45 ^3^
Haemoglobin (g/dl, *mean±SD*)	9.00± 2.06	8.32± 2.27	0.31 ^2^
Plasma cell (pg/dl,*mean ±SD*)	36.88± 25.19	29.50± 11.=87	0.50 ^2^
NF-κB(pg/dl, *mean±SD*)	1.67± 2.12	0.73± 1.10	0.04 ^2^
IL-6 (pg/dl, *mean±SD*)	27.16± 23.69	20.87± 23.85	0.29 ^2^
CRP (mg/l,*mean±SD*)	1.33± 1.35	1.23± 2.18	0.21 ^2^
LDH (U/l±* mean±SD* )	481.86± 818.15	363.71± 144.50	0.63 ^2^
VEGF (pg/dl, * mean±SD* )	387.39± 245.3	245.99± 309.99	0.03 ^2^
TNF-α (pg/dl, * mean±SD* )	22.05± 11.11	19.90± 9.33	0.67 ^2^

Note 1) Mann-Whitney U Test, 2) Independent T-Test, 3) Chi-Square Test MPC, Melphalan-Prednisone-Curcumin; MP, Melphalan-Prednisone-Placebo; ECOG, “Eastern Cooperative Oncology Group;” NF-κB, “Nuclear Factor kappa B”; IL-6, “Interleukin-6”; CRP, “C-reactive protein”; LDH, “Lactic Acid Dehydrogenase”; VEGF, “Vascular endothelial Growth Factor”; TNF-α, “Tumor Necrosis Factor-α”

**Table 2:** Effect of Curcumin in the remission

		**MPC group **	**MP Control**	**Total**
Overall remission	Number % on treatment group	9 (75%)	4 (33.3%)	13 54.2%
Stable disease	Number % on treatment group	3 (25.0%)	8 (66.7%)	11 45.8%
Total	Number	12	12	24

Note: Chi-Square, (X^2^= 6.89, df=1, *p*=0.01)

 Abbreviation: MPC, Melphalan-Prednisone-Curcumin; MP, Melphalan-Prednisone-Placebo

**Table 3:** Effect of curcumin in the transcription factor and cytokine levels

***Variable***	***M*** **onth**	***Level of transcription factor and cytokine ()***		*p*	
		***MCP group***	***MP c*** **ontrol group**		
NF-κB (pg/dL)	1 2 3 4	0.0795 0.4654 0.1294 0.6636	0.0947 0.0468 -.0584 -.0508	0.149 0.133 0.525 0.027	
**IL-6** (pg,dL)	1 2 3 4	2.13 13.91 13.99 19.46	8.50 9.68 6.40 8.30	**0.326 ** **0.954** **0.149** **0.176**	,
VEGF (pg/dL)	1 2 3 4	100.20 76.46 76.04 187.40	18.87 -80.82 3.89 -43.38	0.021 0.013 0.021 0.001	
TNF-α (pg/dL)	4	13.07	2.07	0.004	
CRP (mg/L)	1 2 3 4	0.74 0.62 0.20 0.63	0.81 0.30 -0.31 0.55	0.557 0.074 0.309 0.929	,
LDH (U/L)	1 2 3 4	202.94 220.76 143.36 128.77	36.71 66.81 30.13 75.41	0.736 0.986 0.157 0.008	

Note : Mann Whitney U Test; Friedman Test; IL-6 levels ( MPC group *P*=0.00, MP group *P*=0.19), CRP levels (MPC (*P*=0.01) and MP group (*P*=0.04) MPC, Melphalan-Prednisone-Curcumin; MP, Melphalan-Prednisone-Placebo; NF-κB, “Nuclear Factor kappa B”; IL-6, “Interleukin-6”; CRP, “C-reactive protein”; LDH, “Lactic Acid Dehydrogenase”; VEGF, “Vascular endothelial Growth Factor”; TNF-α, “Tumor Necrosis Factor-α”

**Table 4:** Multivariate Analysis of Curcumin, LDH, VEGF, TNF-α on Remission Status

	**Variable**	***Coefficient (β)***	***SE***	***Wald***	***P***	**OR**	**95% CI**	**R** ^2^
Step 1	Curcumin	-1.135	1.94	0.34	0.56	0.3	0.01-14.33	0.70
	VEGF	-0.003	0.01	0.64	0.42	1	0.99-1.00	
	LDH	0.384	0.26	3.18	0.08	1.5	0.96-1.02	
	TNF-α	-0.034	0.03	1.73	0.19	0.9	1.15-457.7	
	*Constant*	6.658	6.09	1.20	0.27	779		
Step 2	VEGF	-0.004	0.01	1.12	0.29	0.1	0.99-1.00	0.69
	LDH	0.306	0.15	4.40	0.04	1.4	1.02-1.81	
	TNF-α	-0.025	0.02	2	0.16	1	0.94-1.01	
	*Constant*	4.353	4.19	1.08	0.30	77.7		
Step 3	TNF-α	0.297	0.14	4.69	0.03	1.4	1.03-1.76	0.64
	LDH	-0.027	0.02	2.73	0.10	1	0.94-1.01	
	*Constant*	4.363	3.99	1.19	0.28	78.4		

Note*: *multiple logistic regression*, *VEGF, "Vascular endothelial growth factor"; LDH, “Lactic dehydrogenase”; TNF-α, “Tumor necrosis factor-α”

## Discussion

This study proved that there was a difference in overall remission between the MPC and control groups, significantly. The group treated with curcumin has better outcome than the control according to this remission status. Curcumin effects increase overall remission of 75%. Previous studies showed that myeloma patients who took melphalan prednisone had a low overall response rate of less than 50%, with CR below 5% (27, 28). To the best of our knowledge, this is the first report that demonstrated that curcumin has an effect in increasing overall remission in myeloma patients.

﻿Curcumin has anti-inflammatory and anti-cancer effects. Curcumin interferes with multiple cell signaling pathways, including cell cycle (cyclin D1 and cyclin E), apoptosis proliferation (HER-2, EGFR, and AP-1), survival (PI3K/AKT pathway), invasion (MMP-9 and adhesion molecules), angiogenesis (VEGF), metastasis (CXCR-4) and inflammation (NF-κB, TNF, IL-6, IL-1, COX-2, and 5-LOX). Curcumin suppressed initiation, progression, angiogenesis, and metastasis of a variety of tumors. The activity of curcumin has been reported against leukemia, lymphoma, gastrointestinal cancers, genitourinary cancers, breast cancer, ovarian cancer, head and neck squamous cell carcinoma, lung cancer, melanoma, neurological cancers, and sarcoma (29-31). Monoclonal gammopathy of undefined significance (MGUS) and smoldering multiple myeloma (SMM) has a risk of progression to multiple myeloma. The preliminary results showed that patients taking curcumin, paraprotein serum drops between 5% and 30% compared with patients on placebo. Curcumin consumption has the potential risk to slow progression in both MGUS and SMM (13, 32) (13)’(32). Curcumin also affects decreasing transcription factor, NF-κB. ﻿Treatment with curcumin downregulated the expression of NF-κB. Previous studies revealed that curcumin has anti-inflammatory, its activity was induced by various pathways including TNF-α (11, 30, 32, 33). ﻿This study revealed that curcumin affects in decreasing NF-κB level after 4 cycles of treatment and the remission of myeloma patient has a correlation with TNF- levels.

﻿Numerous studies reported that IL-6 promotes the survival and proliferation of multiple myeloma (MM) cells through the phosphorylation of a cell-signaling protein, STAT3 (19). ﻿Interleukin-6 (IL-6) is a cytokine for the defense against acute environmental stress. The pathology in various inflammation and autoimmune disease was caused by persistent dysregulation of IL-6 production diseases(34). Myeloma is a malignancy that is a chronic inflammatory disease (35), it means there is dysregulation of IL-6. This study revealed the decrease of IL-6 level in the group with curcumin. In myeloma patient, the overall survival was significantly different between the low IL-6 and high IL-6 groups, and IL-6 level correlates with the clinical feature and prognosis(36). 

The angiogenic activators important in malignancies are vascular endothelial growth factor (VEGF). VEGF has an impact on developing the new vessel in tumors called neovascularization. The new drug was developed against VEGF like bevacizumab approved by US Federal Drug Administration (37, 38). Our study showed that curcumin affects decreasing VEGF levels. ﻿In this study, we found that curcumin has activities against TNF-α. Previous reports described that curcumin can block the action and production of TNF-α (17). TNF-α is very important in the pathophysiology of human multiple myeloma(39). TNF-α is also highly expressed in tumors and is thought to be pro-angiogenic cytokine. Paradoxically, at it is in higher doses can be used to destroy tumor vascular (37). TNF-α regulates the tumor environment and it has a pleiotropic effect as a cytokine in the process of apoptosis, angiogenesis, inflammation, and immunity(40).

This study showed, that curcumin does not affect decreasing CRP and LDH levels. C-reactive protein (CRP) was the acute inflammation in interacting with the complement system. These proteins increase in inflammation process and are associated with various diseases including hypertension, atherosclerosis, peripheral vascular disease, diabetes, and metabolic disease (41). The level of serum CRP has an association with the number of osteolytic bone lesions (42). The study reported that curcumin has anti-inflammatory through a significant reduction of IL-6, hs-CRP, and MDA levels (42), not CRP. A high level of serum LDH is a poor prognostic factor in patients with malignancies(43). LDH is a useful marker progression in myeloma(44), but also is found in high levels after physical activities. In Chinese elderly patients with myeloma, LDH has an unfavorable prediction for the outcome (45). Curcumin affects the decreasing NF-κB, IL-6, VEGF, and TNF- levels. Curcumin improves overall remission in myeloma patient. Multivariate analysis proved that TNF- correlates with remission. This study revealed that curcumin improves remission through decreasing TNF- level. Previous studies demonstrated that curcumin affected the transcription and angiogenic factors especially TNF-. 

In conclusion **t**he addition of curcumin in myeloma patients treatment with MP regiment will improves the overall remission. Curcumin affects the decrease of NF-κB, IL-6, VEGF, and TNF-α levels. A future study is needed to evaluate the effects of curcumin to the survival and effect of curcumin in combination with another novel agent. 
